# Exploration of SUMO2/3 Expression Levels and Autophagy Process in Fragile X-Associated Tremor/Ataxia Syndrome: Addressing Study Limitations and Insights for Future Research

**DOI:** 10.3390/cells12192364

**Published:** 2023-09-26

**Authors:** Maria Isabel Alvarez-Mora, Glòria Garrabou, Laura Molina-Porcel, Ruben Grillo-Risco, Francisco Garcia-Garcia, Tamara Barcos, Judith Cantó-Santos, Laia Rodriguez-Revenga

**Affiliations:** 1 Biochemistry and Molecular Genetics Department, Hospital Clinic of Barcelona, 08036 Barcelona, Spain; mialvarez@clinic.cat (M.I.A.-M.); barcos@recerca.clinic.cat (T.B.); 2 CIBER of Rare Diseases (CIBERER), Instituto de Salud Carlos III, 08036 Barcelona, Spain; garrabou@clinic.cat (G.G.);; 3 Fundacio de Recerca Clínic Barcelona-Institut d’Investigacions Biomediques August Pi i Sunyer (FRCB-IDIBAPS), 08036 Barcelona, Spain; lmolinap@clinic.cat; 4 Inherited Metabolic Diseases and Muscle Disorders’ Research Laboratory (U722), Cellex-IDIBAPS, Faculty of Medicine and Health Sciences, University of Barcelona, Internal Medicine Department––Hospital Clínic Clinic of Barcelona, 08036 Barcelona, Spain; 5 Alzheimer’s Disease and Other Cognitive Disorders Unit, Neurology Service, Hospital Clinic, 08036 Barcelona, Spain; 6 Neurological Tissue Bank of the Biobanc-Hospital Clinic-FCRB-IDIBAPS, 08036 Barcelona, Spain; 7 Bioinformatics and Biostatistics Unit, Principe Felipe Research Center (CIPF), 46012 Valencia, Spain; rgrillo@cipf.es (R.G.-R.); fgarcia@cipf.es (F.G.-G.)

**Keywords:** *FMR1* premutation, FXTAS, SUMO2/3, p62, SUMOylation, autophagy

## Abstract

Fragile X-associated tremor/ataxia syndrome (FXTAS) is a late-onset neurodegenerative disorder that appears in adult *FMR1* premutation carriers. The neuropathological hallmark of FXTAS is an intranuclear inclusion in neurons and astrocytes. Nearly 200 different proteins have been identified in FXTAS inclusions, being the small ubiquitin-related modifier 2 (SUMO2), ubiquitin and p62 the most highly abundant. These proteins are components of the protein degradation machinery. This study aimed to characterize SUMO2/3 expression levels and autophagy process in human *postmortem* brain samples and skin fibroblast cultures from FXTAS patients. Results revealed that FXTAS *postmortem* brain samples are positive for SUMO2/3 conjugates and supported the idea that SUMO2/3 accumulation is involved in inclusion formation. Insights from RNA-sequencing data indicated that SUMOylation processes are significantly upregulated in FXTAS samples. In addition, the analysis of the autophagy flux showed the accumulation of p62 protein levels and autophagosomes in skin fibroblasts from FXTAS patients. Similarly, gene set analysis evidenced a significant downregulation in gene ontology terms related to autophagy in FXTAS samples. Overall, this study provides new evidence supporting the role of SUMOylation and autophagic processes in the pathogenic mechanisms underlying FXTAS.

## 1. Introduction

Fragile X-associated tremor/ataxia syndrome (FXTAS, OMIM# 300623) is a late-onset neurodegenerative disorder that appears in adult *FMR1* (Fragile X Messenger Ribonucleoprotein 1) premutation carriers (55–200 CGGs). *FMR1* premutation is not infrequent in the general population, affecting approximately 1 out of 800 males and 1 out of 250 females and leading to symptoms of FXTAS in up to 1 in 3000 men older than 50 years [1,2]. Clinical symptoms in FXTAS patients usually begin with an action tremor. After that, different findings may occur and gradually progress, including ataxia and, more variably, loss of sensation in the distal lower extremities and autonomic dysfunction (e.g., impotence, hypertension, and loss of bowel and bladder function). Cognitive deficits are also observed and include memory problems and executive function deficits, with a gradual progression to dementia in some individuals. Some patients also have Parkinsonian symptoms and experience neuropsychological disturbances, including anxiety, reclusive behavior, and irritability or mood liability [3,4,5]. FXTAS shows reduced penetrance and variable clinical expressivity; it is estimated that at least one-third of all *FMR1* premutation carriers will develop FXTAS by the fifth decade of life [6].

As in several other neurodegenerative disorders, the neuropathological hallmark of FXTAS consists of the presence of ubiquitined intranuclear inclusions that are variably distributed throughout the brain [7,8]. FXTAS protein aggregates are distinctively composed of the *FMR1* mRNA and many proteins [9,10]. Although the role of the inclusions in the pathogenesis of FXTAS remains unknown, identification of the proteins sequestered is a key step for understanding how toxicity of *FMR1* premutation alleles occurs. In this regard, nearly 200 different proteins enriched in FXTAS inclusions have been recently identified, being the small ubiquitin-related modifier 2 (SUMO2), ubiquitin and p62/sequestosome-1 (p62/SQSTM1; hereafter designated p62), the most highly abundant ones [11]. These proteins have in common that all of them are components of the protein degradation machinery, depicting the importance that this pathway might have in the pathogenesis of the disease. Based on these results, the authors proposed that inclusions might be repositories of damaged/oxidized proteins, driven by an increase in reactive oxygen species (ROS) and mitochondrial dysfunction, which cannot be efficiently removed because of deficient proteosomal machinery [11]. An increasing body of evidence shows that protein aggregation and inefficient cellular degradation functions (in particular, the autophagy pathway) are functionally interconnected and induce each other during neurodegenerative processes [12,13,14]. In an attempt to provide more insights into the alterations occurring during the neurodegenerative process in FXTAS patients, we characterized the protein expression of SUMO2/3 and the autophagy flux in either or both human postmortem brain samples or skin fibroblast cultures from FXTAS patients. In addition, these findings were further correlated with gene expression data obtained from RNA sequencing analysis of both tissues.

## 2. Material and Methods

### 2.1. Postmortem Human Brain Samples

Human samples of deep-frozen postmortem prefrontal cortex, hippocampus and cerebellar vermis were obtained from two female FXTAS brain donors and two sex and age-matched controls with no clinical evidence of FXTAS or neuropathological finding of any main neurodegenerative diseases but mild changes commonly observed in the ageing brain (Table 1). FXTAS patients had a clinical diagnosis before death. They were neuropathologically confirmed based on the postmortem identification of a variable amount of intranuclear ubiquitin-positive inclusions in neuronal and glial cells [15]. All donors were of Caucasian origin. Brain samples were obtained from the Hospital Clinic-FRCB-IDIBAPS Brain Bank under approved protocols of the Ethical Committee (Hospital Clinic Barcelona, Barcelona, Spain).

### 2.2. Skin Fibroblast Cultures

Skin fibroblast cultures were obtained from four unrelated FXTAS patients (one female and three males) and four control individuals (two females and two males). All patients were recruited from fragile X syndrome families. FXTAS group encompasses patients who meet criteria in any of the three categories of involvement: definite, probable, and possible [3] (Table 2).

Fibroblasts were collected and cultured as previously described [22]. All participants provided written informed consent for testing and the use of their phenotypic/clinic and genetic data. The study was approved by the Ethics Committee of the Hospital Clinic of Barcelona, following the guidelines of the Helsinki Declaration (2013).

### 2.3. SUMO2/3 Immunohistochemistry Examination

Immunohistochemistry was performed on 5 um formalin-fixed and paraffin-embedded brain tissue sections using BOND-MAX Automated Immunohistochemistry Stainer and a BOND Polymer Refine Detection system (Leica Biosystems Melbourne Pty Ltd., Melbourne, Australia). Primary antibodies used were SUMO2/3 1:800 (Abcam, Cambridge, UK, ab3742), anti-Ubiquitin 1:5000 (P4D1; Cell signaling, Danvers, MA, USA) and anti-p62 1:500 (3/P62 LCK LIGAND, BD Bioscience, San Jose, CA, USA). The presence of SUMO2/3 inclusions in both control subjects and subjects with FXTAS was determined blind to the subjects’ genetic information and clinical and pathological diagnosis.

### 2.4. SUMO2/3 Protein Quantification

To quantify SUMO2/3 protein levels by western blot analysis, whole protein lysates from the brain and fibroblasts were obtained from all the samples recruited in the study. Cell lysates were subjected to SDS-PAGE and electroblotted. Proteins were visualized by immunostaining with antibody against SUMO2/3 1:1000 (Abcam, ab3742) and β-actin 1:5000 (A5441, Merck/Sigma-Aldrich, Madrid, Spain). Colorimetric detection (1708235 Opti-4CNTM Substrate Kit, Bio-Rad, Hercules, CA, USA) and ImageJ software were used for densitometry analysis of protein expression.

### 2.5. Functional Enrichment Analysis

RNA sequencing data from the FXTAS patients (skin fibroblasts and postmortem prefrontal cortex brain samples) were gathered for the present work, and additional samples were available as part of previous studies from our group (unpublished data). In particular, RNAseq was performed from skin fibroblasts (6 FXTAS patients and three controls) and postmortem prefrontal cortex brain samples (12 controls and 3 *FMR1* premutation carriers) in Centro Nacional de Análisis Genómico (CNAG, Barcelona, Spain). Functional enrichment analysis was performed on the differential expression profiles between patients and control samples in the Centro de Investigación Principe Felipe (Valencia, Spain). The Gene Set Analysis (GSA) method implemented in the mdgsa R package with the Gene Ontology (GO) (http://geneontology.org/, accessed on 6 June 2023) and Reactome databases (https://reactome.org/, accessed on 6 June 2023), as functional annotations were applied [23]. This method detects significantly up-or down-regulated blocks of functionally related genes in lists of genes ordered by differential expression. Given that many functional terms are simultaneously tested, the test results are corrected for multiple testing to obtain an adjusted *p*-Value. The *p*-Values were corrected using the Benjamini & Hochberg method (1995) [24], and pathways with an adjusted *p*-Value < 0.05 were considered significantly deregulated. The GSA method uses a logistic regression model, meaning that significant and positive log odds ratios (LOR) indicate up-regulated pathways, while significant and negative LOR indicate down-regulated pathways.

### 2.6. Autophagic Flux Analysis

To monitor autophagic flux a time-course experiment was carried out in skin fibroblast cultures from FXTAS patients and controls treated with Bafilomycin A1 (Merck/Sigma-Aldrich, Madrid, Spain) reagent. Bafilomycin A1 is a reagent to monitor autophagosome synthesis since it inhibits autophagosome-lysosome fusion [25]. Three different times of Bafilomycin A1 treatment were established: untreated cells (basal condition), 4 and 8 h after treatment. Afterwards, total protein content was obtained through SYPRO Ruby Protein Blot Stain, according to the manufacturer’s protocol (ThermoFisher Scientific, Madrid, Spain), to further perform western blotting analysis. Blots were probed with antiSQSTM1/p62 (ab155686, Abcam) and anti-LC3B (#2775, Cell Signaling Technology, Danvers, MA, USA) antibodies. The p62 protein targets specific cargoes for degradation; thus, its measurement reflects the account of autophagy substrates. On the other hand, the LC3BII protein is used as a reporter of autophagy activity since it is located in the autophagosome membrane. Densitometric analysis (Image Quant TL 8.1 Software, GE Healthcare, Madrid, Spain) was used to quantify the intensity of signals. Results were expressed as p62 and LC3BII protein levels normalized by the total cell protein content (using SYPRO staining).

### 2.7. Immunocytochemistry for Autophagosome Evaluation

To perform fibroblast immunocytochemistry, 5000 cells were counted using the Scepter 2.0 Handheld Automated Cell Counter pipette (Merck) and seeded in a 16-well glass slide (Nunc™ #178599 Lab-Tek^®^ Chamber Slide™, Austin, TX, USA) at 37 °C with 5% CO_2_ for 24 h. Cells were fixed with 4% paraformaldehyde for 15 min and permeabilized with 0.1% Triton X-100 in a blocking solution (1% bovine serum albumin). Degradation of autophagolysosomes was blocked by adding 100 nM Bafilomycin A1 (Sigma-Aldrich^®^ #B1793 SIGMA, St. Louis, MO, USA) for 6 h. Autophagosomes were stained with anti-LC3 pAB (MBL International^®^ #PM036, Woburn, MA, USA) and secondary marked through the donkey anti-rabbit Alexa Fluor^®^ 488 IgG antibody (Life Technologies Europe, Bleiswijk, Netherlands). Counterstain with DAPI was performed for nuclei staining (DAPI Fluoromount-G^®^ #0100-20, Southern Biotech, AL, USA). Images were taken with a Zeiss LSM 880 laser scanning confocal system (63×).

### 2.8. Statistical Analysis

Results were expressed as means ± the standard error of the mean (SEM). For comparisons of the means, the statistical significance of the differences was examined using the parametric independent *t*-test, or the non-parametric Mann–Whitney U test with commercially available software (SPSS-PC, version 19; SPPSS Inc., Chicago, IL, USA). Significance was accepted for asymptotic bilateral *p*-Values < 0.05.

## 3. Results

### 3.1. FXTAS Postmortem Brain Samples Are Positive for SUMO2/3 Conjugates

**Figure 1:**
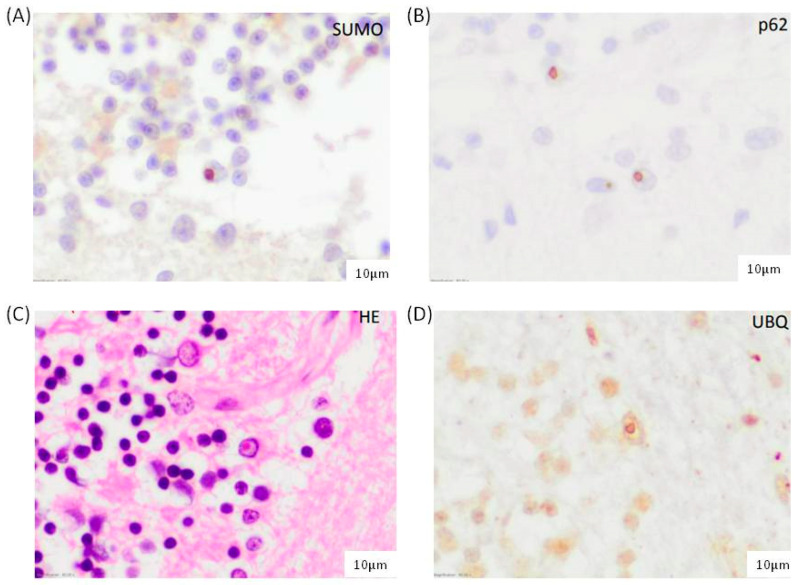
Characterization of intranuclear inclusions in the cerebellar vermis of FXTAS-2 subject observed with (**A**) SUMO2/3, (**B**) p62, (**C**) hematoxylin-eosin, and (**D**) ubiquitin immunostaining Scale bar: 10 µm.

To evaluate SUMO2/3-conjugated proteins at the cellular and subcellular level, SUMO2/3 immunohistochemistry analysis was performed with anti-p62 and anti-ubiquitin antibodies in FXTAS postmortem brain samples. SUMO2 and SUMO3 are 95% homologous and are detectable with the same antibody. Since cortical neurons and astrocytes are described to accumulate FXTAS inclusions [8], we analyzed the prefrontal cortex, hippocampus and cerebellar vermis of patients and controls. As shown in Figure 1A, SUMO2/3 staining in patients with FXTAS showed intranuclear inclusions in astrocytes and neurons, which were not observed in control subjects. We observed a mild frequency of inclusions in the frontal cortex of subject #2 (FXTAS_brain_2), moderate in the gray matter of the cerebellum, especially around the Purkinje cell layer, and moderate and frequent in the CA4 and CA3 regions of the hippocampus. However, in subject #1 (FXTAS_brain_1), inclusions were isolated in the frontal cortex and hippocampus, absent in the cerebellum, as previously described [15].

Furthermore, the morphology, frequency, and distribution of conjugated SUMO2/3 aggregates were identical to those of intranuclear inclusions positive for ubiquitin and p62 described in FXTAS brain cells (Figure 1B–D). As previously reported, these results indicated the accumulation of SUMO2/3 conjugated proteins in FXTAS patients and support that SUMO2/3 accumulation is involved in inclusion formation.

### 3.2. Western Blot Analysis of SUMO2/3 Expression in Brain and in Skin Fibroblasts from FXTAS Patients

To quantify the increase of SUMO2/3 in FXTAS brain nuclei, western blot analyses were carried out in tissue lysates from the prefrontal cortex, hippocampus, and cerebellar vermis of FXTAS patients (*n* = 2) and controls (*n* = 2) as well as in skin fibroblasts lysates from FXTAS patients (*n* = 4) and controls (*n* = 4). No differences were seen in FXTAS patients compared to control individuals in none of the tissues analyzed.

### 3.3. SUMOylation Related Pathways Are Significantly Altered in Brain and in Skin Fibroblasts Samples from FXTAS Patients

Although the levels of SUMO2/3-conjugated proteins were not different between patients and controls, we investigated SUMOylation pathways in expression profiles obtained from RNA sequencing data from FXTAS brain and fibroblast patients. Functional enrichment analysis using the Reactome database indicated that SUMOylation processes are significantly upregulated in both tissues’ FXTAS gene expression profiles (Table 3). When analyzing expression levels of genes related to these pathways, 37 genes were identified as significantly deregulated in FXTAS brain samples (Table 4). None of the skin fibroblast samples from FXTAS patients reached a significant *p*-Value.

### 3.4. Fibroblasts from FXTAS Patient’s Evidence Accumulation of p62 Protein Levels and Increased Autophagy Processes

**Figure 2:**
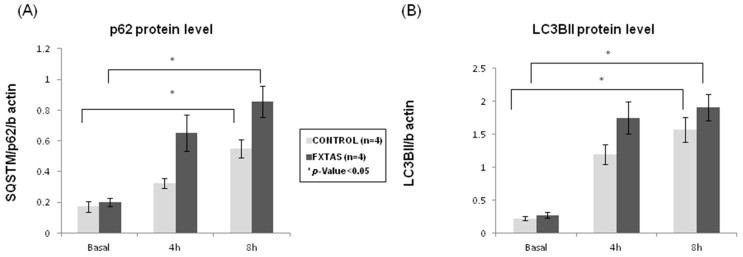
Autophagic flux assessment in FXTAS patients and control skin culture fibroblasts. (**A**) p62 and (**B**) LC3BII protein level at basal (0 h) and under bafilomycin A1 treatment (4 or 8 h). The results are expressed as means ± standard error of the mean (SEM).

It is known that SUMOylation cooperates with the ubiquitin–proteasome system to maintain the homeostasis of proteins (e.g., [26]) Multiple studies showed that SUMO2/3-conjugated and ubiquitinated proteins accumulate once the proteasome system is inhibited [27,28]. In this scenario and to provide better insight into the relationship between SUMO and protein degradation in FXTAS patients, we further evaluated the autophagic flux and activity in skin fibroblast cultures. The experiment measured p62 and LC3BII protein levels by western blot in a time-course experiment treating cells with bafilomycin A1. When comparing p62 and LC3BII levels between FXTAS and controls, no statistically significant differences were observed at basal conditions (*p* = 0.75 for p62 protein level; *p* = 0.65 for LC3BII protein levels) (Figure 2).

Nevertheless, after bafilomycin A1 cells treatment, which blocks autophagosome degradation and thus allows the measurement of autophagosome synthesis, a gradual and significant increase was found between basal condition and 8 h bafilomycin A1 treatment for p62 protein levels (*p* = 0.04 for controls; *p* = 0.02 for FXTAS patients) (Figure 2A). Although this increment was found in both groups, when comparing the fold change between different experiment conditions, FXTAS patients consistently showed a 30% higher increase of p62 than controls. On the other hand, when analyzing LC3BII results, despite there was also a significant increase of LC3BII levels between basal condition and 8 h (*p* = 0.01 for controls; *p* = 0.007 for FXTAS patients), the FXTAS group presented a 20% higher increase than control when comparing fold change between basal condition and 4 h treatment (Figure 2B). The same comparison at 8 h treatment did not show differences in FXTAS patients, indicating that at 8 h of bafilomycin A1 treatment, the autophagic flux may have reached a plateau. Overall, these results suggest that skin fibroblasts from FXTAS patients are accumulating autophagic cargo (p62 labeling) in a higher proportion than control cells and that the autophagic flux is enhanced, probably to mediate the degradation of damaged cell organelles and proteins. Nevertheless, it cannot fully be discarded that the enhanced autophagic flux is a consequence of a primary alteration of the process per se.

**Figure 3:**
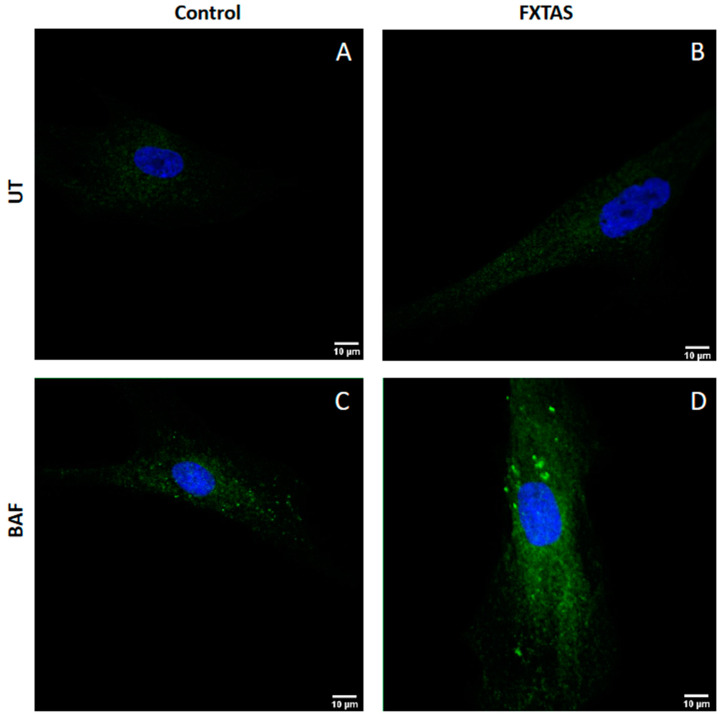
Visualization of autophagosomes. Representative images of autophagosome formation in untreated fibroblast (UT) and 6 h treatment with 100 nM bafilomycin fibroblast (BAF) obtained by confocal microscopy (63×). (**A**) Untreated fibroblast from control individuals. (**B**) Untreated fibroblast from FXTAS patients. (**C**) 6 h treatment fibroblast from control individuals. (**D**) 6 h treatment fibroblast from FXTAS patients.

Finally, to visualize changes in autophagosome number in FXTAS cells, a time-course LC3BII immunohistochemistry experiment was performed in skin fibroblast cultures. Two different times of bafilomycin A1 treatment were established (untreated cells and 6 h). As shown in Figure 3, the accumulation of LC3BII signal in FXTAS patients after treatment is higher than in controls.

### 3.5. Regulation of Autophagy Is Significantly Altered in Brain Samples and in Skin Fibroblasts from FXTAS Patients

Gene set analysis was carried out for the Gene Ontology (GO) terms related to autophagy. We found that the biological process GO term related to “regulation of macroautophagy GO:0016241” was significantly under-represented in FXTAS patients compared to controls in both, brain, and skin fibroblast tissues (Table 3). Table 4 summarizes the 24 significantly deregulated genes related to this biological process identified in FXTAS brain samples. None reached a significant *p*-Value in skin fibroblast samples from FXTAS patients.

## 4. Discussion

Proteostasis refers to the processes involved in maintaining the correct protein conformation, concentration, and subcellular location [29]. It plays a pivotal role in many neurodegenerative diseases where aggregation of specific proteins into protein inclusions is a characteristic. The removal of these aggregates together with misfolded and/or damaged proteins or organelles is essential to assure cell viability, especially in non-dividing cells such as neurons. Although it is still no clear whether protein inclusions are neuroprotective or cytotoxic, consequence or causal of the disease, what seems irrefutable is that their formation is closely related to an ineffective or overwhelmed protein homeostasis network (reviewed in [30]).

Having shown that SUMO2/3 and p62 are within the highly abundant proteins in FXTAS inclusions [11] and that they have a role in cellular strategies of protein quality control [28,31], this study aimed to characterize SUMOylation and autophagy pathways in FXTAS patients. Firstly, SUMO2/3 distribution was examined by immunohistochemistry in the postmortem prefrontal cortex, hippocampus, and cerebellar vermis of FXTAS patients and controls. Similar to those previously described, results showed that SUMO2/3 stained within inclusions, as SUMO2/3 immunoreactivity overlapped with p62 and ubiquitin staining (Figure 1). However, when quantifying SUMO2/3 by western blot in total brain lysates from FXTAS patients and controls, and in consonance with previous reports, no differences were obtained. This may be due to the low intranuclear inclusions in our cases and, on the other hand, to the use of total brain lysates, which do not differentiate cellular types or cellular compartments. These results reinforce the idea that SUMO2/3 conjugates may get diluted in whole tissue lysates, as previously pointed out [11]. No statistically significant differences between FXTAS patients and controls were obtained when quantifying SUMO2/3 in whole lysates from skin fibroblasts (*p* > 0.05).

Secondly, a functional enrichment analysis was performed using transcription profiling data obtained from RNA sequencing in both postmortem brain samples and skin fibroblast from FXTAS patients and controls. Upregulation of SUMOylation pathways was found in both tissues (Table 3), which seems to agree with the increase in SUMO2/3 conjugation activity observed in the postmortem prefrontal cortex of FXTAS patients. The co-localization of SUMO2/3 with neuronal inclusions and the upregulation of SUMOylation pathways suggest a potential role of SUMOylation in FXTAS pathogenesis.

Next, the autophagy flux was monitored in skin culture from FXTAS patients. Autophagy is a lysosome-dependent degradation process capable of clearing protein aggregates and damaged organelles [32]. Compared to controls, FXTAS patients showed a consistent 30% higher accumulation of p62 in all time points of the experiment and a 20% higher accumulation of LC3BII protein levels at 4 h treatment. This LC3BII accumulation was maintained at 8 h treatment, suggesting that the autophagic flux in FXTAS patients is enhanced and saturated. The accumulation of autophagosomes in FXTAS patients was consistently evidenced by LC3BII immunohistochemical analysis (Figure 3). Accordingly, autophagy-related biological processes were deregulated in expression profiles obtained from FXTAS brain and skin fibroblasts RNA sequencing data (Table 3). These results suggest that FXTAS cells are under stress trying to eliminate the excess of autophagy substrate. Bioenergetic collapse leading to cellular stress has been reported in FXTAS patients. We and others have previously described reactive oxygen species (ROS) accumulation, mitochondrial dysfunction and abnormalities of calcium signaling as altered pathways in FXTAS pathogenesis (e.g., [22,33,34]).

This current study supports that the autophagic flux is altered in FXTAS patients. Only one article on autophagy and FXTAS has been published, using a Drosophila model expressing retinal CGG90 [35]. In this work, authors used rapamycin, an efficient inducer of autophagy, which has been shown to have a protective effect against many neurodegenerative diseases, decreasing the accumulation of mutant proteins. In the FXTAS Drosophila model, enhancing autophagy was ineffective in suppressing neurotoxicity [35]. This observation is in line with the enhanced and exhausted autophagic flux herein described and might explain why, in the FXTAS Drosophila model, enhancing autophagy was insufficient to alleviate neurotoxicity. To an endpoint, overactive autophagy causes an alteration of the cell protein homeostasis, leading to cell death (e.g., [36]). The p62 protein and SUMO2/3 are both proteins implicated in the ubiquitin-proteasome system, and their levels are stimulated in a stress-dependent manner [37]. It has been described that protein inclusions may enhance the efficiency of aggregate clearance, presumably by facilitating interaction with the lysosomal and autophagic pathways [38]. Thus, the elevated levels of SUMO2/3 and p62 protein found in FXTAS patients, together with the upregulation of SUMOylation-related pathways and enhanced autophagic flux, might be reflecting a protective cellular response to the toxic effect of misfolded proteins accumulated in the inclusions [38]. We found that the deregulation of these pathways was more pronounced in brain tissue, where inclusions are predominantly found than in skin fibroblasts from FXTAS patients. Perhaps these structures develop only when a threshold of misfolded protein is exceeded, and since neurons are non-dividing cells, their ability to clear out is less than dividing cells such as skin fibroblasts.

This study has some limitations that need to be addressed to ensure the viability and robustness of the findings. First, the small sample size might explain the lack of significance in some analyses and increase the margin of error. Second, the difference in sex distribution of samples, which may introduce some bias. The fact that only brain samples from female FXTAS patients have been studied might attenuate the abnormal levels of SUMO2/3 and p62 proteins since a portion of the cells will be expressing the normal *FMR1* allele. On the contrary, in skin fibroblast, most FXTAS samples were obtained from male patients. Despite the difference in sex distribution, the results obtained were in consonance in both tissues, which reinforces the reliability of the findings. The final limitation refers to the use of skin fibroblasts, which might not constitute an appropriate model due to clonal selection and drift in culture. However, the fact that we have shown the same pathways altered in both skin fibroblasts and brain tissues reinforces their use as a robust research model and the veracity of the herein-reported results.

In conclusion, this study contributes new evidence that relates the processes of SUMOylation and autophagy with the pathology of FXTAS. By overcoming the above-discussed limitations, the reported results can significantly contribute to unravel the molecular mechanisms of *FMR1* premutation toxicity and potentially uncover novel therapeutic avenues for this complex neurodegenerative disorder.

## Figures and Tables

**Table 1:** Clinical, molecular and neuropathological findings of brain samples from patients and controls included in the study.

ID Sample	Sex	Age at Disease Onset (Years)	Age at Death (Years)	Expanded *FMR1* (CGG)n	Frontal Cortex SUMO Intranuclear Inclusions	Hippocampal SUMO Intranuclear Inclusions	Cerebellar SUMO Intranuclear Inclusions	Clinical Symptoms	Relevant Neuropathological Findings
FXTAS_brain_1	Female	73	78	77	Isolated GII in white matter	Isolated GII inclusion in dentate gyrus	0	Dementia, tremor, ataxia	Low AD-NC (A1B1C0), AGD (stage I), e, VBI
FXTAS_brain_2	Female	70	95	60	mild GII and NII in gray matter	Moderate NII and GII in CA4/CA3	Moderate GII and NII in Grey matter	Hypoacusia, tremor, ataxia, dementia	High AD-NC (A3B3C3), LBD (Braak 1), VBI
Control_brain_1	Female	-	81	-	none	none	none	Control	Low AD-NC (A3B1C2), LATE-NC (stage 2)
Control_brain_2	Female	-	90	-	none	none	none	Control, stroke	PART (definitive, Braak II), VBI

AD-NC = Alzheimer’s disease neuropathological changes [16]; AGD = argyrophilic grain disease [17]; GII = glial intranuclear inclusions, LATE-NC = Limbic-predominant age-related TDP-43 encephalopathy neuropathological changes [18]; LBD = Lewy Body Disease [19,20]; NII = Neuronal intranuclear inclusions, PART = primary age-related tauopathy [21]; VBI = Vascular Brain Injury.

**Table 2:** Clinical and molecular characterization of skin fibroblasts cultures of patients and controls included in the study.

	Sex	Age (Years) at Skin Biopsy	CGG Repeat Size	Clinical Diagnosis
FXTAS_1	Male	70	65	Ataxia and tremor
FXTAS_2	Male	62	95	Ataxia and tremor
FXTAS_3	Female	53	30/149	Intention tremor and memory defects
FXTAS_4	Male	66	98	Ataxia, tremor and dementia
Control_1	Female	82	21/30	control
Control_2	Male	57	30	control
Control_3	Male	86	30	control
Control_4	Female	68	30/32	control

**Table 3:** Selected Reactome pathways and Gene Ontology (GO) terms deregulated in FXTAS patients compared to controls.

		Brain Sample			Fibroblast Sample		
PATH_ID	PATH_NAME	Size	padj	LOR	Size	padj	LOR
R-HSA-3108232	SUMO E3 ligases SUMOylate target proteins	146	0.003515	0.3012	151	0.0004707	0.3446
R-HSA-2990846	SUMOylation	152	0.005241	0.2856	157	0.0002597	0.349
R-HSA-3108214	SUMOylation of DNA damage response and repair proteins	71	0.0000425	0.5717	70	0.009913	0.3955
R-HSA-4570464	SUMOylation of RNA binding proteins	42	0.04191	0.4147	41	0.0321	0.4362
R-HSA-4085377	SUMOylation of SUMOylation proteins	31	0.03038	0.5127		NS	
R-HSA-3232142	SUMOylation of ubiquitinylation proteins	34	0.04552	0.4545		NS	
GO:0016241	Regulation of macroautophagy	48	0.00108	−0.6426	47	0.01945	−0.4926

LOR: Logarithm of the Odds Ratio. NS: not significant.

**Table 4:** Significant deregulated genes in *postmortem* brain samples of FXTAS patients.

Genes Related to SUMOylation Processes			Genes Related to Autophagy Processes		
Gene	Fold Change	padj	Gene	Fold Change	padj
*THRB*	−2.231	0.001	*ATP6V1H*	−1.486	0.004
*SATB1*	−1.555	0	*UCHL1*	−2.233	0
*CHD3*	−1.401	0.013	*ATP6V1G2*	−2.104	0
*PPARGC1A*	−1.374	0.005	*CDK5R1*	−2.059	0.002
*SATB2*	−1.168	0.008	*ATP6V1A*	−2.03	0
*SUMO3*	−0.876	0.027	*ATP6V1B2*	−2.004	0
*SAE1*	−0.728	0.016	*CDK5*	−1.872	0
*NUP93*	−0.669	0.048	*PRKAA2*	−1.774	0
*SUMO1*	−0.659	0.029	*ATP6V1C1*	−1.739	0
*CETN2*	−0.593	0.039	*ATP13A2*	−1.096	0.012
*NRIP1*	−0.57	0.027	*ATP6V1D*	−1.008	0.01
*NUP50*	−0.557	0.027	*ATP6V0A1*	−0.939	0
*ING2*	−0.548	0.019	*ATP6V0C*	−0.936	0.008
*TP53BP1*	−0.523	0.008	*PRKACA*	−0.732	0.016
*NSMCE3*	−0.519	0.023	*ATP6V0D1*	−0.717	0.033
*INCENP*	−0.409	0.047	*VPS26B*	−0.712	0.001
*RAE1*	−0.344	0.048	*MAPK8*	−0.,708	0.007
*TRIM28*	0.292	0.035	*VPS35*	−0.669	0.047
*SP3*	0.329	0.041	*UBQLN2*	−0.608	0.001
*SENP1*	0.343	0.036	*UBQLN1*	−0.451	0.003
*SEC13*	0.367	0.024	*SNX5*	0.319	0.008
*ZBED1*	0.435	0.009	*HDAC6*	0.436	0.032
*PARP1*	0.462	0.027	*SH3GLB1*	0.542	0.016
*NUP43*	0.473	0.015	*ATP6V0E1*	0.991	0.003
*AAAS*	0.515	0.013			
*STAG1*	0.555	0.002			
*SMC3*	0.569	0.007			
*NUP160*	0.606	0.032			
*NSMCE1*	0.638	0.002			
*PCNA*	0.638	0.033			
*NUP37*	0.645	0.036			
*NSMCE4A*	0.74	0.033			
*STAG2*	0.809	0.018			
*TP53*	0.911	0.02			
*NFKBIA*	1.03	0.046			
*SP100*	1.165	0.005			
*CDKN2A*	1.522	0.028			

## Data Availability

The analyzed data sets generated during the study are available from the corresponding author upon reasonable request. All data underlying the findings described in the manuscript are fully available upon request and without restriction.
